# A straightforward procedure to build a non-toxic relaxometry phantom with desired T1 and T2 times at 3T

**DOI:** 10.1007/s10334-024-01166-7

**Published:** 2024-05-11

**Authors:** Victor Fritz, Sabine Eisele, Petros Martirosian, Jürgen Machann, Fritz Schick

**Affiliations:** 1 Section of Experimental Radiology, Department of Diagnostic and Interventional Radiology, University of Tübingen, Hoppe-Seyler-Str. 3, 72076 Tübingen, Germany; 2 Institute for Diabetes Research and Metabolic Diseases of the Helmholtz Centre Munich at the University of Tübingen, Tübingen, Germany; 3 German Center for Diabetes Research (DZD), Neuherberg, Germany

**Keywords:** MRI Phantom, Relaxometry, T1, T2, 3T, Soy Lecithin

## Abstract

**Objective:**

To prepare and analyze soy-lecithin-agar gels for non-toxic relaxometry phantoms with tissue-like relaxation times at 3T.

**Methods:**

Phantoms mimicking the relaxation times of various tissues (gray and white matter, kidney cortex and medulla, spleen, muscle, liver) were built and tested with a clinical 3T whole-body MR scanner. Simple equations were derived to calculate the appropriate concentrations of soy lecithin and agar in aqueous solutions to achieve the desired relaxation times. Phantoms were tested for correspondence between measurements and calculated T1 and T2 values, reproducibility, spatial homogeneity, and temporal stability. T1 and T2 mapping techniques and a 3D T1-weighted sequence with high spatial resolution were applied.

**Results:**

Except for the liver relaxation phantom, all phantoms were successfully and reproducibly produced. Good agreement was found between the targeted and measured relaxation times. The percentage deviations from the targeted relaxation times were less than 3% for T1 and less than 6.5% for T2. In addition, the phantoms were homogeneous and had little to no air bubbles. However, the phantoms were unstable over time: after a storage period of 4 weeks, mold growth and also changes in relaxation times were detected in almost all phantoms.

**Conclusion:**

Soy-lecithin-agar gels are a non-toxic material for the construction of relaxometry phantoms with tissue-like relaxation times. They are easy to prepare, inexpensive and allow independent adjustment of T1 and T2. However, there is still work to be done to improve the long-term stability of the phantoms.

## Introduction

Phantoms serve as substitutes for biological tissue and are indispensable tools in quantitative magnetic resonance imaging (qMRI) research. Gels with known (or even adjustable) tissue-like T1 and T2 relaxation properties are useful for research, especially for developing and testing new pulse sequences and post-processing techniques [1, 2] as human volunteers are not always available for long test sessions. Since medical device-grade phantoms with desired relaxation properties are not present at all research sites, a protocol for flexible, homemade phantoms could be very useful. Ideally, the materials used to make the phantoms should be inexpensive, non-toxic, and easy to work with.

In particular, relaxometry phantoms that mimic tissue-like relaxation times attract the interest of researchers. Relaxation times T1 and T2 are intrinsic tissue properties that depend on tissue composition and microenvironment [3]. Changes in native T1 and T2 values are known as sensitive indicators of various pathologies, including cancer, cardiovascular abnormalities, and brain diseases [4]. To verify accurate and precise T1 and T2 measurements with MRI equipment, the availability of suitable test objects with predefined values is essential.

Interestingly, there are only few approaches to the fabrication of phantoms with tissue-like relaxation times T1 and T2. Relaxometry phantoms proposed so far are typically two-component mixtures consisting of a gelling agent (e.g. agarose, agar) doped with paramagnetic salt (e.g. MnCl_2_, NiCl_2_, GdCl_3_) [5–16]. Here, the paramagnetic salt generally serves as a T1-modifier and the gelling agent as a T2-modifier. Knowing the relaxivities (r_1_,r_2_) of each component, one can design a phantom material that has the desired relaxation times. The required concentrations of the respective substances can be calculated according to the following formulas described by Tofts et al. [8, 9]:

1 $${C}_{a}= \frac{R2-R{2}_{w}-({r}_{2}^{\left(b\right)}/{r}_{1}^{\left(b\right)})(R1-R{1}_{w})}{{r}_{2}^{\left(a\right)}-({r}_{2}^{\left(b\right)}/{r}_{1}^{\left(b\right)}){r}_{1}^{(a)}}$$

2 $${C}_{b}= \frac{R1-R{1}_{w}-({r}_{1}^{\left(a\right)}/{r}_{2}^{\left(a\right)})(R2-R{2}_{w})}{{r}_{1}^{\left(b\right)}-({r}_{1}^{\left(a\right)}/{r}_{2}^{\left(a\right)}){r}_{2}^{(b)}}$$

where C_a_ and C_b_ are the concentrations of component a and b, r_1_^(a,b)^ and r_2_^(a,b)^ are the relaxivities of component a and b, R1_w_ and R2_w_ are the relaxation rates of pure water, and R1 and R2 are the relaxation rates to be achieved.

It has been shown that those phantoms can be reproducibly produced and exhibit high temporal stability [7–9]. However, paramagnetic salts are toxic, which complicates the production, handling and disposal of these phantoms. Furthermore, the presence of paramagnetic salts can strongly affect the magnetic susceptibility of the phantom material [17]. This could lead to adverse effects, especially for gradient echo sequences, as undesirable magnetic field inhomogeneities occur depending on the geometry and composition of the phantom. Therefore, research is underway to find alternative, non-hazardous substances that can replace paramagnetic salt as a T1 modifier and allow phantoms to be produced without safety and toxicity issues [18].

In this context, Sękowska et al. [19] presented MRI phantoms based on non-toxic detonation diamond nanoparticles (DND). The phantoms composed of agar, carageen, and DND particles suspended in dimethyl sulfoxide have been shown to successfully mimic the relaxation times of liver tissue. However, the data from the study suggests that phantoms with T1 values above 950 ms cannot be fabricated, at least not with the fabrication method presented—which is a disadvantage, as many tissues (e.g. gray matter, kidney, spleen, etc.) have T1 values in the range of 1000–2000 ms and therefore cannot be covered.

Another relatively new and promising approach is the use of soy lecithin in MRI phantoms. Soy lecithin is a naturally occurring emulsifier that has recently been found to alter the relaxation times and diffusion properties of water [20, 21]. In addition, soy lecithin is inexpensive, readily available and non-toxic, making it ideal for phantom manufacturing. Therefore, it is obvious to use and test soy lecithin for the preparation of relaxometry phantoms, which was also the motivation for this work.

In the present work, soy-lecithin-agar gels were prepared and evaluated as an alternative phantom material for the construction of relaxometry phantoms with tissue-like relaxation times. Special attention was paid to an understandable presentation of the phantoms’ development and fabrication process. Test phantoms mimicking the relaxation times of different tissue types were evaluated for their correctness (agreement between measured and targeted values), reproducibility and temporal stability.

## Methods

### Study design

Before phantom fabrication can begin, relaxivities r_1_ and r_2_ of soy lecithin and agar have to be determined (see Eqs. 1 and 2). For this purpose, pure aqueous soy lecithin solutions and pure agar gels of different concentrations (0, 1, 2, 3, 4, 5%) were prepared and examined using T1- and T2 mapping techniques.

Furthermore, it has to be ensured that the two substances are compatible and retain their effect even when mixed. A change in relaxivity as a function of the concentration of the other substance would make it impossible to apply simple Eqs. (1, 2) for determination of concentrations and thus produce correct phantoms. At least, the T1 modifier should have stable longitudinal relaxivity r_1_ and the T2 modifier should have stable transverse relaxivity r_2_. To verify this, relaxivities of soy-lecithin were measured in the presence of different concentrations of agar (1%, 2%, 3%, 4%), while agar-relaxivities were measured in the presence of different concentrations of soy lecithin (1%, 2%, 3%, 4%).

After the preliminary experiments, test phantoms were designed to match the relaxation times T1/T2 of different tissues: grey matter (1820 ms/99 ms [3]), white matter (1084 ms/69 ms [3]), kidney cortex (1142 ms/76 ms [22]), kidney medulla (1545 ms/81 ms [22]), spleen (1328 ms/61 ms [22]), liver (812 ms/42 ms [3]), and muscle (1295 ms/34 ms [22]). Using the previously determined relaxivities and Eqs. 1 and 2, the appropriate concentrations of soy-lecithin and agar were calculated to achieve the desired T1 and T2 values. For the relaxation rates of pure water, the values R1_w_ = 2950 ms and R2_w_ = 2000 ms were used (determined by several preliminary measurements).

The phantoms were tested for correctness (agreement between measured and target values), reproducibility and temporal stability. For this, all phantoms were prepared three times, independently on different days and examined on the day of preparation and 4 weeks after preparation. Possible mold growth was monitored by visual inspection of the samples during the 4-week study period. In addition, the samples were checked for homogeneity and the absence of air bubbles. Air bubbles are problematic because they cause magnetic field distortions that lead to susceptibility artifacts [15].

### Preparation of the phantoms

**Fig. 1:**
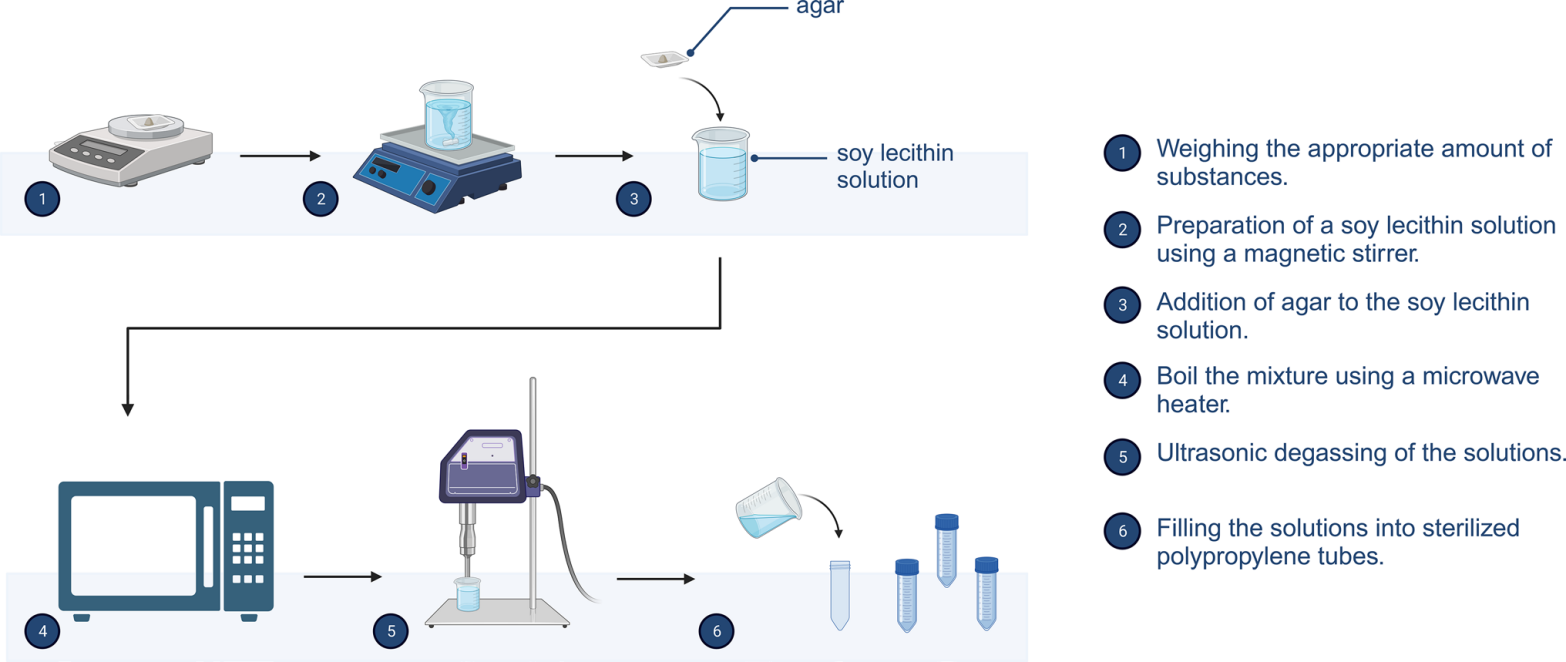
Schematic representation of the manufacturing process of the soy-lecithin-agar phantoms. Created with BioRender.com

Phantoms were prepared as follows (Fig. 1): First, the appropriate amount of soy lecithin (Carl Roth, Karlsruhe, Germany) was dissolved in demineralized water (Carl Roth, Karlsruhe, Germany) under magnetic stirring at 650 rpm for 10 min. Agar (Agar powdered pure, food grade, PanReac-AppliChem-ITW Reagents, Darmstadt, Germany) was then stirred into the soy lecithin solution and the mixture was boiled using a microwave heater until the solution was clear and homogeneous. The solutions were then filled into sterilized polypropylene tubes (50 ml, Greiner Bio-One, Frickenhausen, Germany) and cooled to room temperature for gelation. High viscosity solutions were sonicated with an ultrasonic homogenizer (Hielscher Ultrasonics, Teltow, Germany) to remove air bubbles prior to solid gel formation. Pure aqueous soy lecithin solutions and agar gels of different concentrations (0, 1, 2, 3, 4, 5%) were prepared by the same procedure without addition of the other substance.

**Fig. 2:**
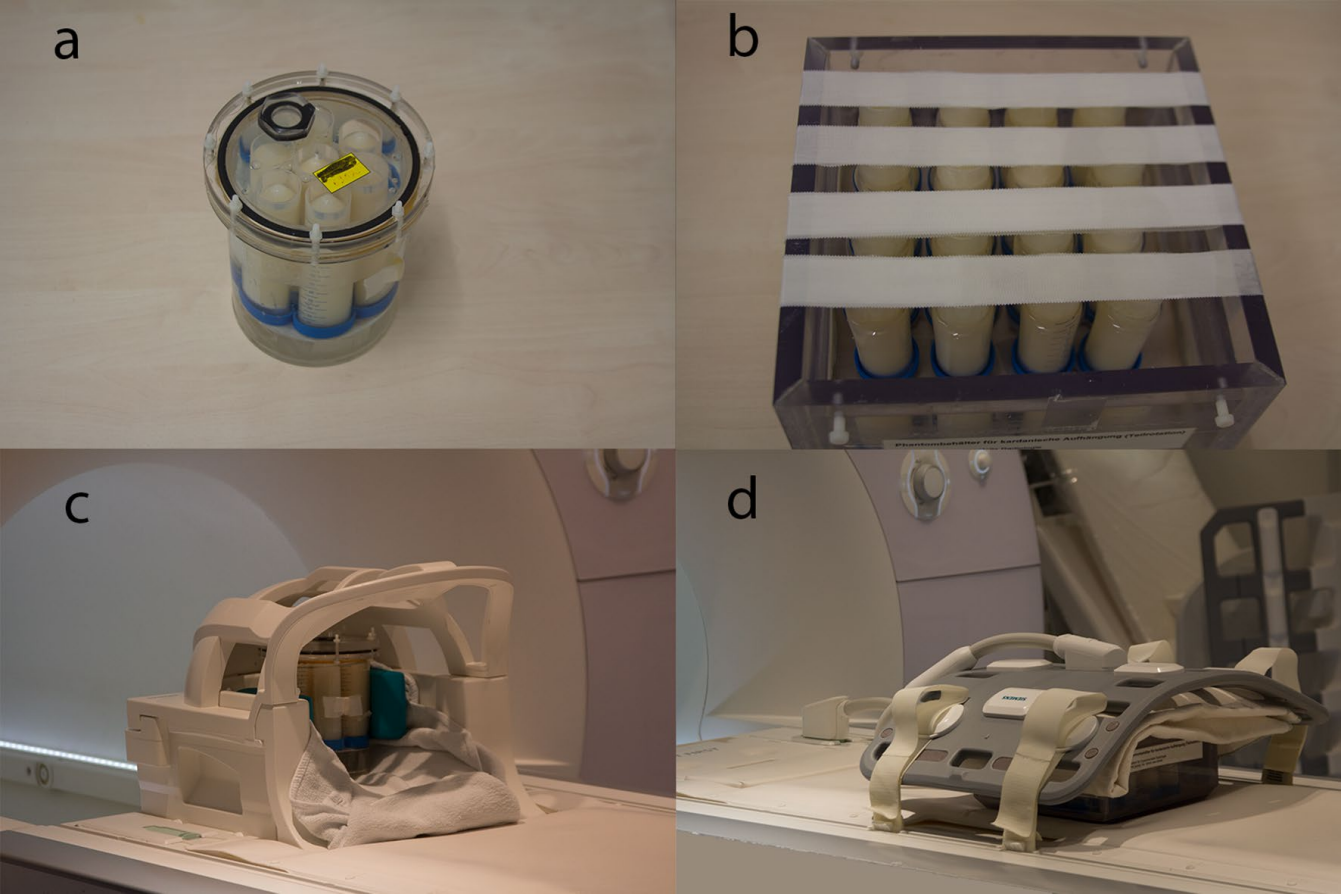
Photographs of the water-filled sample tube housings: **a** cylindrical housing with 7 sample tubes **b** larger square housing with 16 sample tubes. Photographs of the measurement setup on a clinical 3T whole-body MR scanner: **c** the cylindrical housing with 7 sample tubes was scanned using a 20-channel head coil **d** the square housing with 16 sample tubes was scanned using an 18-channel body array coil

For the measurements, the samples were fixed in water-filled MR-compatible housings (Fig. 2a–b): The measurements to determine the individual relaxivities of soy lecithin and agar as well as for the final test phantoms were performed using a cylindrical housing that can hold up to 7 tubes. In contrast, due to the large number of samples, the compatibility measurements of soy lecithin and agar were carried out using a larger square housing that can hold up to 16 phantoms.

All phantoms were stored in the scanner room for at least 6 h before measurements to ensure that the temperature of the samples could stabilize and adapt to the ambient temperature. Between measurements, the samples were stored in the dark in a laboratory cabinet at a room temperature of approx. 21 °C.

### Data acquisition and analysis

Measurements were performed on a clinical 3.0 Tesla whole-body MR scanner (MAGNETOM Prisma^fit^, Siemens Healthcare, Erlangen, Germany) with a 20-channel head coil at 21 °C ± 0.5 °C.

Relaxivity measurements of soy lecithin and agar in the mixture (compatibility measurements) were performed using an 18-channel body array coil, as the square housing containing all samples did not fit into the head coil. The corresponding measurement setups are shown in Fig. 2c–d. All data were processed and analyzed offline using in-house developed software (MATLAB, MathWorks, Natick, MA).

T1 and T2 measurements were performed with the following parameters: matrix = 128 × 128, FOV = 200 × 200 mm, slice thickness = 5 mm, number of slices = 1, slice in coronal plane, positioned in the center of the samples.

T1 was measured using a single slice inversion recovery turbo spin echo pulse sequence (IR-TSE) with TR of 10,000 ms and TE of 9.9 ms. Images were acquired for 9 different TIs in the range of 25–6400 ms (logarithmically equally spaced). T1 maps were calculated from the acquisitions with multiple TIs by pixel-wise monoexponential fitting of signal intensities (SI): *SI* = *SI*_*0*_* (1–a exp(-TI/T1)* + *exp(-TR/T1))* [23]*.*

T2 was measured in the same slice using a multi-echo CPMG spin echo pulse sequence with a TR of 6000 ms and 32 TEs ranging from 10 to 320 ms (equally spaced). Since soy lecithin has a relatively small effect on T2, the T2 decay for the pure soy lecithin solutions (without agar) was sampled for longer TEs in the range of 50–1600 ms (equally spaced). T2 maps were calculated on a pixelwise basis by monoexponential fitting of the measured SI’s: *SI* = *SI*_*0*_* exp(-TE/T2)* + *c* [22]. All signal values were noise corrected before fitting.

Relaxation times (T1, T2) and relaxation rates (R1 = 1/T1, R2 = 1/T2) of each sample were determined from circular regions of interest in the calculated parametric maps. Relaxivities were calculated from the linear regression of the relaxation rates on the concentration of the substance: R1,2 = r_1,2_ *·* [concentration] + c. The slope of the line represents the relaxivity r_1,2_.

A 3D T1-weighted gradient echo sequence (VIBE) with high spatial resolution was applied to examine the phantoms for homogeneity and the absence of air bubbles. Acquisition parameters include: TR = 6.3 ms, TE = 2.46 ms, FOV = 256 × 256, spatial resolution = 0.5 × 0.5x0.5 mm, number of slices = 10, coronal planes.

## Results and discussion

**Fig. 3:**
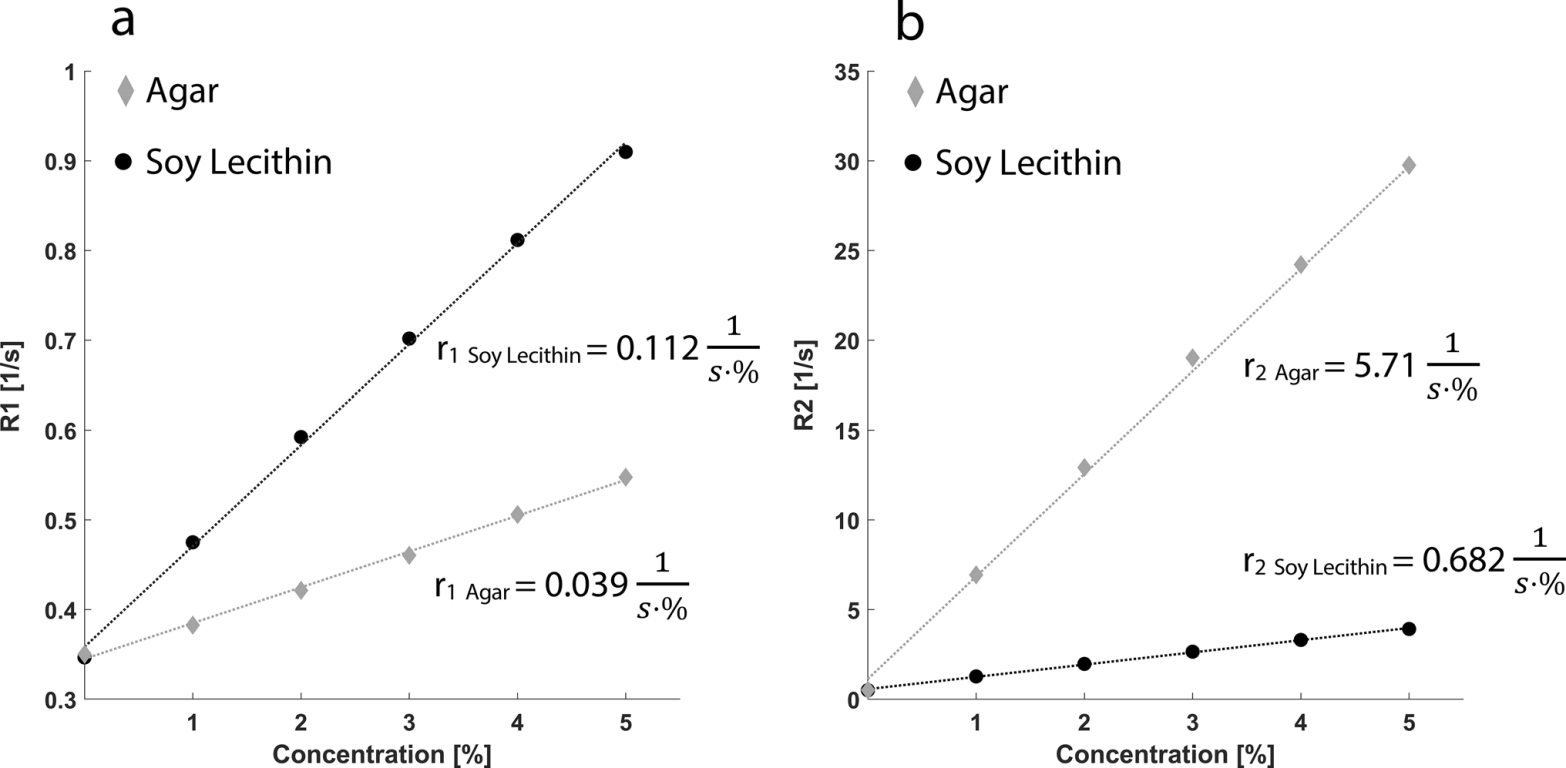
Relaxation rates R1 (**a**) and R2 (**b**) of aqueous solutions as a function of soy lecithin and agar concentration, respectively

First, the relaxation rates R1 and R2 of pure aqueous soy lecithin solutions and pure agar gels were measured at concentrations up to 5%. For both soy lecithin and agar, the relaxation rates showed a linear correlation with concentration (Fig. 3). Relaxivities r_1_ and r_2_ calculated using a linear fit gave r_1,lecithin_ = 0.112 s^−1^·wt.%^−1^ (R^2^ = 0.99), r_1,agar_ = 0.039 s^−1^·wt.%^−1^ (R^2^ = 0.99), r_2,lecithin_ = 0.68 s^−1^·wt.%^−1^ (R^2^ = 0.99), and r_2,agar_ = 5.71 s^−1^·wt.%^−1^ (R^2^ = 0.99), which is in good agreement to previous work [20].

Secondly, soy lecithin and agar were found to retain their effect even when mixed. Table 1 lists the relaxivities of soy lecithin as a function of agar concentration. It becomes clear that both, r_1_ and r_2_, hardly change in the presence of agar. Relaxivities showed little variation in the range of 0.112–0.119 s^−1^·wt.%^−1^ for r_1_ and 0.68–0.77 s^−1^·wt.%^−1^ for r_2_, respectively. Similarly, agar relaxivities remained nearly constant in the presence of soy lecithin (Table 2). Relaxivities varied between 0.027–0.039 s^−1^·wt.%^−1^ for r_1_ and 5.60–5.88 s^−1^·wt.%^−1^ for r_2_, respectively.

**Table 1:** Relaxivities r_1_ and r_2_ of soy lecithin in the presence of different agar concentrations. To measure the relaxivities of soy lecithin, the soy lecithin concentration was varied between 0%–4% (in steps of 1%) while the agar concentration was kept constant

	Soy lecithin			
Agar [%]	r_1_ [s^−1^·wt.%^−1^]	R^2^	r_2_ [s^−1^·wt.%^−1^]	R^2^
0	0.112	0.99	0.68	0.99
1	0.117	0.99	0.70	0.99
2	0.117	0.99	0.68	0.98
3	0.119	0.99	0.77	0.96
4	0.114	0.99	0.74	0.90
Mean	0.116	–	0.71	–

**Table 2:** Relaxivities r_1_ and r_2_ of agar in the presence of different soy lecithin concentrations. To measure the relaxivities of agar, the agar concentration was varied between 0%–4% (in steps of 1%), while the soy lecithin concentration was kept constant

	Agar			
Soy lecithin [%]	r_1_ [s^−1^·wt.%^−1^]	R^2^	r_2_ [s^−1^·wt.%^−1^]	R^2^
0	0.039	0.99	5.71	0.99
1	0.033	0.95	5.88	0.99
2	0.032	0.97	5.60	0.99
3	0.027	0.98	5.83	0.99
4	0.033	0.99	5.87	0.99
Mean	0.033	–	5.78	–

After determining the relaxivities and confirming the compatibility of soy lecithin and agar, the preparation of test phantoms mimicking organ related relaxation times was performed. For the relaxivities, the mean values r_1,lecithin_ = 0.116 s^−1^·wt.%^−1^, r_1,agar_ = 0.033 s^−1^·wt.%^−1^, r_2,lecithin_ = 0.71 s^−1^·wt.%^−1^, and r_2,agar_ = 5.78 s^−1^·wt.%^−1^ (see Table 1 and 2) were used in the following.

Substituting the calculated relaxivities into Eqs. 1 and 2 yields the following relationship between agar or soy lecithin concentration and desired relaxation times:

3 $${C}_{Agar}= \frac{180}{T2 [ms]} - \frac{1100}{T1 [ms]} + 0.28$$

4 $${C}_{Lecithin}= \frac{8930}{T1 [ms]} - \frac{51}{T2 [ms]} - 3$$

**Fig. 4:**
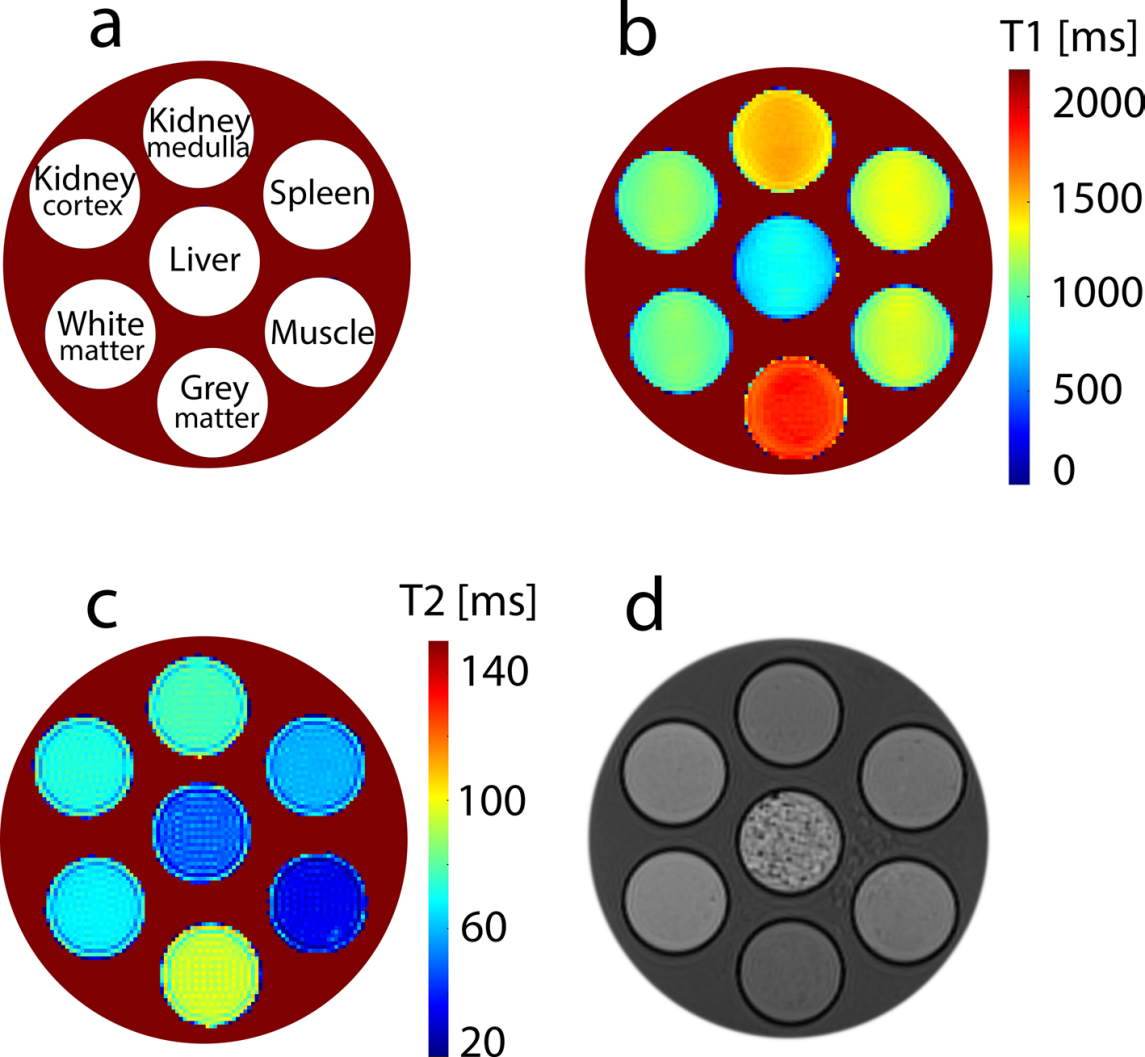
Parametric maps of test phantoms mimicking relaxation times of different tissues (grey matter, white matter, kidney cortex, kidney medulla, spleen, muscle, liver). **a** Representation of the positions of the test phantoms in the cylindrical measuring housing. **b** Parametric map of T1 times. **c** Parametric map of T2 times. **d** 3D T1-weighted image with high spatial resolution of test phantoms mimicking relaxation times of different tissues. With the exception of the liver phantom, all phantoms were homogeneous and showed little to no air bubbles. In contrast, the liver phantom had an inhomogeneous or brittle structure with many air bubbles

Using these equations, samples were produced with relaxation times corresponding to the T1 and T2 times published in the literature for gray and white matter, kidney cortex and medulla, spleen, muscle, and liver. Table 3 provides an overview of the concentrations of soy lecithin and agar used, as well as the targeted and measured T1- and T2 times. The corresponding T1- and T2 maps can be seen in Fig. 4a–c. Good agreement was found between the measured and targeted relaxation times. The largest deviations occurred for the liver phantom, where the percent deviation from the T1 set point was 2.7% and from the T2 set point was 32.5%. For all other phantoms, the percentage deviations from target relaxation times were less than 3% for T1 and less than 6.5% for T2, which is comparable to phantoms made with paramagnetic salts [15, 16]. In addition to the acceptable correspondence between calculated relaxation times and measured values, the reproducibility of the manufacturing process and resulting relaxation times T1 and T2 seems also sufficient for most applications, as shown by the relatively small standard deviations across the three batches (Table 3).

**Table 3:** Overview of the concentrations of soy lecithin and agar used and the targeted and measured T1 and T2 times of the test phantoms. The mean value and the standard deviation over the three measured phantom batches are shown

	T1 [ms]		T2 [ms]		Soy lecithin [%]	Agar [%]
	Target	Measured	Target	Measured		
Gray matter	1820	1850 ± 12	99	93 ± 3	1.39	1.49
White matter	1084	1093 ± 4	69	66 ± 2	4.50	1.87
Kidney cortex	1142	1159 ± 10	76	72 ± 2	4.15	1.69
Kidney medulla	1545	1539 ± 17	81	75 ± 3	2.15	1.79
Spleen	1328	1318 ± 23	61	58 ± 1	2.89	2.40
Muscle	1295	1262 ± 16	34	34 ± 1	2.40	4.72
Liver	812	834 ± 16	42	56 ± 6	6.78	3.21

3D T1-weighted images with high spatial resolution showed that the samples, with the exception of the liver phantom, were quite homogeneous and contained little to no air bubbles (Fig. 4d). Only the liver phantom had an inhomogeneous or brittle structure and showed significant air bubbles. Even degassing with ultrasound could not remove those air bubbles. This can be explained by the comparatively high concentrations of soy lecithin and agar required to prepare the liver phantom (see Table 3). The combination of high soy lecithin (> 6%) and high agar (> 3%) concentration results in a very viscous mixture, which in turn favors the entrapment of air bubbles that form during the heating process. The resulting low homogeneity of the sample could also be the reason for the relatively high deviation between measured and target T2 time (32.5%) in the liver phantom. This indicates that gels mimicking tissues that have both short T1- and T2 times (eg. Liver, myocardial tissue) are problematic, as high concentrations of agar and soy lecithin are required. Evacuation of the surroundings of highly viscous gels could help to reduce or avoid air bubbles. However, this would require additional equipment and an additional preparation step.

**Fig. 5:**
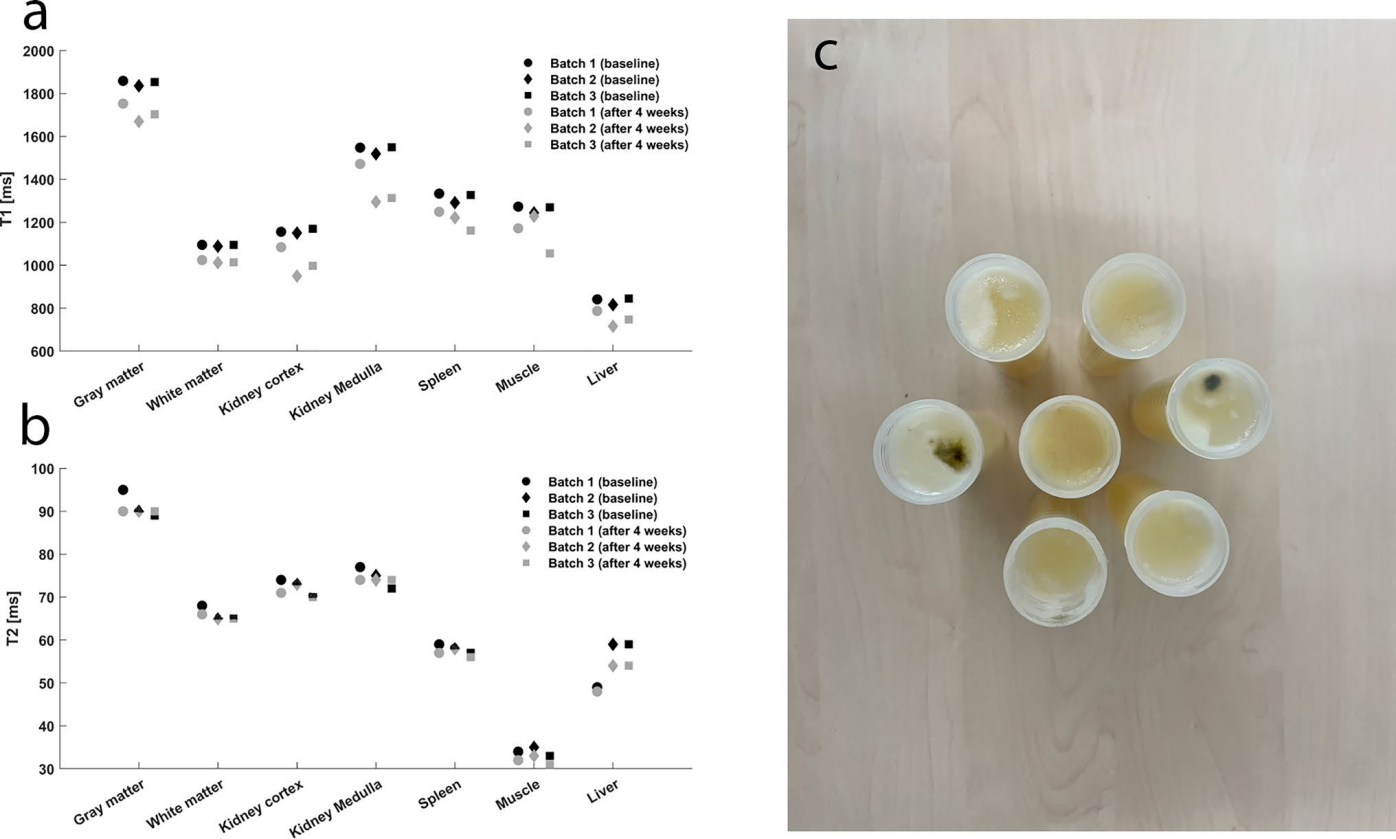
To evaluate the temporal stability of the test phantoms, T1 and T2 measurements were repeated after 4 weeks under the same conditions. **a** Comparison of T1 times measured at baseline and after 4 weeks. **b** Comparison of T2 times measured at baseline and after 4 weeks. **c** Photograph of the test phantoms (batch 1) after a storage period of 4 weeks—mold growth is clearly visible on some phantoms

The temporal stability of the phantoms was evaluated after a storage period of 4 weeks. Unfortunately, the phantoms were unstable over time, which was particularly reflected in the T1 times. Across all batches, the T1 times of all phantoms decreased significantly compared to the first measurement (Fig. 5a). The changes in T2 times were not quite as pronounced, but here too most phantoms showed a deviation from the initial measurement (Fig. 5b).

One possible reason for the altered relaxation behavior of the phantoms could be biodegradation by microorganisms. Without suitable additives, agar gels provide an ideal nutrient medium for fungi and bacteria [24], which biodegrade the phantom material over time and thus also alter MR properties. This could also be observed macroscopically on some phantoms by means of mold growth (Fig. 5c).

The limited temporal stability of the soy-lecithin-agar phantoms is a major drawback compared to previously proposed phantoms using paramagnetic salts for T1 or T2 modification. Solutions with inorganic substances are stable for a long time without significant change in relaxation times [7]. This feature is very important when phantoms are employed for reproducibility measurements in multicenter studies where measurements are carried out over several weeks or even months.

To increase the biostability of soy-lecithin-agar phantoms and thus prevent microbial growth, preservatives such as fungicides and or bactericides can be used. It has been shown that the addition of these agents can maintain the stability of agar phantoms for up to 2 years [25]. However, most of the effective agents are quite toxic, which militates their use as it is contrary to the motivation of this study (production of relaxometry phantoms without toxic or questionable substances). Other preservatives such as citric acid or sodium sulfite, which are mainly used in the food industry [26], are harmless, but change the pH of the medium. Changing the pH would also be unfavorable since pH affects not only the properties of agar gels but also the micelle formation of soy lecithin molecules [27–29]. Soy lecithin is an amphiphilic molecule that forms micelles in aqueous solutions, the number, type and shape of which depend on various environmental parameters (pH, temperature, etc.) [27, 28]. This means that a preservative that affects the pH also changes the microstructure of the phantoms and thus their MR properties. It is still a challenge to find a suitable non-toxic preservative that will ensure the stability of the phantoms without compromising the effect of soy lecithin and agar. A number of systematic measurements are needed that are beyond the scope of this work but are planned for future studies. Another potential way to increase temporal stability is sterilization of the water or autoclaving the entire gels or UV irradiation of the phantoms. In this context, sensitivity of the organic substances to heat must be considered.

The temperature dependence of relaxation properties of the proposed gels has not been investigated so far. Knowledge of the temperature dependence can be important to account for temperature-related measurement deviations in practical usage of the phantoms. Furthermore, there is unpredictable dependence of relaxation on the magnetic field strength. In this study, the soy lecithin agar phantoms were examined at a field strength of 3T only. Further studies are needed to investigate their properties at higher and lower field strengths. Effects of different approaches for relaxometry (pulse sequences and data processing) is also an interesting area of research. It is a well-documented issue that relaxation times measured with different methods can vary considerably, even in the same subjects examined with the same MRI system [23, 30]. Further work will investigate whether similar variations in the relaxation times measured with different relaxometry approaches can also be observed in the soy-lecithin-agar phantoms.

While the soy-lecithin-agar phantoms effectively mimic desired relaxation times and can serve as valuable tools for testing relaxometry methods, it is important to emphasize that they do not replicate the complexity of biological tissue. For example, they do not accurately reproduce tissue properties such as relaxation anisotropy, which is observed in highly anisotropic tissues such as white matter. Previous studies have shown that T1 and T2 relaxation times in white matter are angle-dependent due to the orientation of axon fibers in the B_0_ magnetic field [31–34]. This anisotropic nature of relaxation times cannot be mimicked by structurally homogenous phantoms.

## Conclusion

This work shows that soy-lecithin-agar gels represents an alternative phantom material for the construction of relaxometry phantoms with tissue-like relaxation times, thus expanding the toolbox of qMRI-research. Soy-lecithin agar gels are inexpensive, easy to prepare, and allow independent adjustment of T1 and T2 without marked susceptibility effects. With the presented manufacturing process, the relaxation times of almost all tissues can be mimicked, and without the use of toxic and/or paramagnetic substances.

Nevertheless, there are still open questions regarding the long-term stability and the temperature dependence of the phantoms which will be addressed in future studies.
